# Patients and Their Beauty Sleep

**Published:** 1914-01-10

**Authors:** 


*To the Editor of* The Hospital.

### Patients and their Beauty Sleep.

*To the Editor of* The Hospital.

Sir,—In answer to the Note in last week's Hospital *re* the early waking of ward patients, may I offer my opinions for the defence of having ward patients awakened between the early hours of 5 a.m. and 6 a.m.? It is so that the hospital may be in working order, everything neat and tidy by at least 9.30 a.m. If the patients were not awakened to be washed before 7 a.m., the following would be the order of the morning's work :—

7 a.m.—Wash all patients (this with the aid of the day nurses will take about an hour).

8 a.m.—Breakfast.

8.30 a.m.—Make beds, and leave patients comfortable.

9.30 a.m.—Probationers sweep the wards; staff nurses take temperatures and see to medicine bottles, etc.

10 a.m.—Probationers dust; staff nurses put on quilts and do four-hourly work.

10.30 a.m.—Probationers clean bathrooms and lavatories.

Thus, by 10.30 a.m. at the very earliest, is the ward in a fit condition to receive a doctor, or for a patient to have a dressing done.

I have in my mind an average ward of twenty-four beds, and perhaps a side ward. Now, this time-table leaves little room for unforeseen emergencies, which in every hospital are always occurring. Thus it would often be 11 a.m. before the nurse (or, in the case of a medical school, a student) could begin "the dressings." In a busy ward these will take three hours or more; they would have to be stopped while the patients were having their dinner, and again when the nurse had hers, and often the 2 o'clock work would be due before the daily dressings were finished; thus delaying them till the middle of the afternoon. What about the patients' "after-dinner sleep," which many of them enjoy? Again, the ward would never be tidy or quiet, and some patients would be waiting the greater part of the day to have their dressings done. To my mind—perhaps because I have worked so long in the old way—everything would be "topsy-turvy." I do not think the patients lose much sleep by being awakened at the early hour. It must be remembered that they have settled down for the night, all lights being out, by 8 p.m. The majority are asleep by 9 p.m.; therefore, they have had eight hours' sleep by 5 a.m., and any sleep they may have lost may be made up during the day. I have learned by experience that often the patient who has had a restless night, after being washed and made comfortable, sometimes as early as 4 a.m., has settled into a nice quiet sleep. The majority of hospital patients belong to the working class, and which of them, when well and at work, are accustomed to and *can* sleep till 8 a.m.? Of course, if there was a larger nursing staff than most hospitals have, the morning's work could be done much quicker; but will our charities supply enough money for this increase? I can assure the precocious correspondent of eleven years that the night nurses do not look upon the washing of the patients as a game at all; and I am sure not one of them would be sorry to have that work taken off their shoulders.—I am, Sir, yours, etc.,

Sister.

January 3, 1914.

[The above letter raises new points in, rather than disposes of, the issue, and we invite further expressions of opinion on this administrative question.—Ed.]

